# Ca Signaling in B Cells

**DOI:** 10.1100/tsw.2010.219

**Published:** 2010-11-16

**Authors:** Taras Lyubchenko

**Affiliations:** Department of Medicine, University of Colorado Denver School of Medicine, USA

**Keywords:** calcium signaling, B lymphocytes, B cell receptor, CD21, immune synapse, anergy, autoimmunity

## Abstract

An increase in intracellular Ca concentration is one of the major initial steps in B-cell activation that occurs within minutes after antigen receptor (BCR) engagement. In recent years, significant advances have been made in characterizing molecular mechanisms of Ca signaling in lymphocytes, although the majority of work was done on T cells. This mini-review discusses several underexplored areas of Ca signaling in B cells: (1) Ca signaling in immune synapse and multifaceted Ca responses within a single cell, (2) source of Ca involved in Ca-dependent protein phosphorylation events and the role of store-operated influx, (3) role of BCR coreceptors in Ca signaling, and (4) Ca signaling and maintenance of B-cell tolerance and clinical significance of Ca signaling alterations.

The role of intracellular $Ca^{2+}$ increases in B-lymphocyte activation induced by antigen receptor (BCR) ligation has been actively studied since the late 1970s. It is well established that in B cells, similar to other nonexcitable cells types, $Ca^{2+}$ serves as a universal second messenger required for signal transduction[1,2,3,4] and that intracellular stores as well as extracellular environment serve as sources of $Ca^{2+}$[5]. Dynamic characteristics of BCR-induced $Ca^{2+}$ responses (amplitude, time course, etc.) are important in the activation of specific transcription factors that regulate immune functions, cell differentiation, proliferation, or cell death[6,7,8]. The majority of work on $Ca^{2+}$ signaling in immune cells has been focused on T lymphocytes and these studies have proven that $Ca^{2+}$ release-activated channels (CRAC) comprise the major influx mechanism and that the transmembrane proteins STIM and *Orai* play key roles in this process[9,10,11]. An identical/very similar CRAC influx mechanism highly selective for $Ca^{2+}$ over other cations (which is a characteristic feature of CRAC[12]) and regulated by STIM was also described in primary murine B lymphocytes[13]. STIM1 and STIM2 are novel transmembrane $Ca^{2+}$ regulatory proteins recently identified and implicated as playing a key role in CRAC-mediated $Ca^{2+}$ influx in lymphocytes[10,14]. In contrast to T cells, the role of STIM1 in antigen receptor–mediated $Ca^{2+}$ signaling in primary B cells and its subcellular localization has not been studied in great detail. Although the presence of STIM1 in B cells was suggested early on[15,16], most of the current knowledge about STIM/*Orai*-mediated influx comes from non–B-cell studies, including T cells, insect cells, and fibroblasts. This mechanism is believed to be evolutionarily conserved and to include B lymphocytes, whereby STIM directly monitors the $Ca^{2+}$ concentration in the endoplasmic reticulum (ER) lumen. These possibilities are discussed in an excellent review[17], along with potential new roles for distinct $Ca^{2+}$mobilization profiles in individual primary B-cell subsets and nuclear translocation of transcription factors as downstream targets of $Ca^{2+}$ mobilization. Recent findings of mutant analysis based on structure–function experiments in chicken DT40 cells suggested that constitutive dynamic movement of STIM1 in the ER subcompartments is obligatory for subsequent depletion-dependent redistribution of STIM1 into puncta underneath the plasma membrane and activation of store-operated channels (SOC)[18]. It has also been demonstrated that the coupling of $Ca^{2+}$ store release to $I_{crac}$ channel activation in DT40 chicken B cells requires tonic activity of Lyn and Syk kinases, and that the action of kinases on $I_{crac}$ activation does not arise from control of the expression level of STIM1 and *Orai1* proteins[19]. Smyth et al.[20] examined $Ca^{2+}$-store–dependent reversal of STIM1 localization in HEK293 cells and demonstrated that SOC $Ca^{2+}$ entry is tightly coupled to formation of STIM1 puncta, and both SOC and puncta formation involve a dynamic and reversible signaling complex. Expression of STIM1 and STIM2 has been reported in CD3+/CD4+-, CD3+/CD8+-, and CD19+ murine lymphocytes[21]. However, recent reports suggest that, unlike STIM1, STIM2 has a smaller role in T-lymphocyte signaling[22]. Given a considerable level of similarities between BCR and TCR signaling mechanisms, STIM2 may not play a major role in B cells as well.

However, CRAC is not the only mechanism of $Ca^{2+}$ delivery in lymphocytes. Other mechanisms of $Ca^{2+}$ homeostasis regulation include well-characterized sarco-endoplasmic reticulum $Ca^{2+}$ ATPases (SERCA)[23] and plasma membrane $Ca^{2+}$ ATPases (PMCA)[24] that transport $Ca^{2+}$ into intracellular stores or outside the cell, respectively; plasma membrane $Na^{+}/Ca^{2+}$ exchange pump[25] and mitochondrial $Ca^{2+}$ transport systems[26]; passive $Ca^{2+}$ diffusion routes through inositoltriphosphate from ER stores into the cytosol[27] and simply through the plasma membrane[28]; membrane potential–modulated channels, such as voltage-gated and $Ca^{2+}$-activated $K^{+}$ channels[29,30] and transient receptor potential melastatin-related (TRPM) family of channels that depolarize the membrane[31,32]; transient receptor potential vanilloid receptors (TRPV)[33] and nonselective $Ca^{2+}$-permeable transient receptor potential channels (TRPC)[34].

One important feature of the CRAC-mediated mechanism is that intracellular $Ca^{2+}$ concentration increases as a result of influx from extracellular media through SOC triggered by a release of relatively small quantities of $Ca^{2+}$ from intracellular stores (Fig. 1). Regulatory mechanisms connecting store depletion with the opening of transmembrane channels have been studied and IP3 has been implicated in the regulation of membrane channels through conformational changes induced by depletion of intracellular $Ca^{2+}$ stores[35]. More recent studies revealed key molecular details of this process in T lymphocytes and established the role of the above-mentioned STIM1/2 and *Orai* proteins as molecular sensors for intracellular $Ca^{2+}$ store depletion and membrane pore unit, respectively. These mechanisms are discussed in great detail in two excellent reviews[36,37] and are beyond the scope of this manuscript.

A number of signaling molecules are involved in the generation of BCR-triggered $Ca^{2+}$ increases in B cells: CD45[38], CD19 and CD21[39,40], STAT3[41], FcγRIIb[42], Btk[43], Syk[44], acetylcholine[45], PCKδ[46], BLNK[47], Zap-70[48], c-Abl[49], c-Myc[50] and others. Non–voltage-gated $Ca^{2+}$ channels with L-type characteristics can also be activated by BCR ligation[51]. Inhibitory phosphatases such as CD22 have also been shown to play a regulatory role in $Ca^{2+}$ signaling, as BCR-triggered influx is enhanced in CD22-deficient B cells[52]. In addition, a number of BCR-specific features of $Ca^{2+}$ signaling have been recently described with mechanisms involving signal amplification through CD20/CD81, PLCγ2/IP3R/STIM1/CRAC, and BCR/cADPR/RYR3/CRAC pathways, as well as modulation pathways that involve CD22, FcγRIIb, SHIP, and SHP1/2 (reviewed in Feske[53]).

Many other aspects of $Ca^{2+}$ signaling in B cells have also been characterized. It was demonstrated that nonselective cation channels may be involved in BCR-independent $Ca^{2+}$ increases in B cells due to shear stress[54] and hypotonicity[43,55,56]. Certain SOC properties of B-cell $Ca^{2+}$ influx in response to BCR-independent stimulation with thapsigargin were also demonstrated[57]. In addition, other BCR-independent stimuli such as oxidant stress[43,58] and peroxide[38] have been shown to elevate intracellular $Ca^{2+}$ in B cells. Lipid raft disruption was found to enhance the release of $Ca^{2+}$ from intracellular stores, suggesting that rafts may sequester early signaling events that down-regulate $Ca^{2+}$ influx[59]. Syk and Lyn also play a role in BCR-independent $Ca^{2+}$-induced apoptosis in B cells after

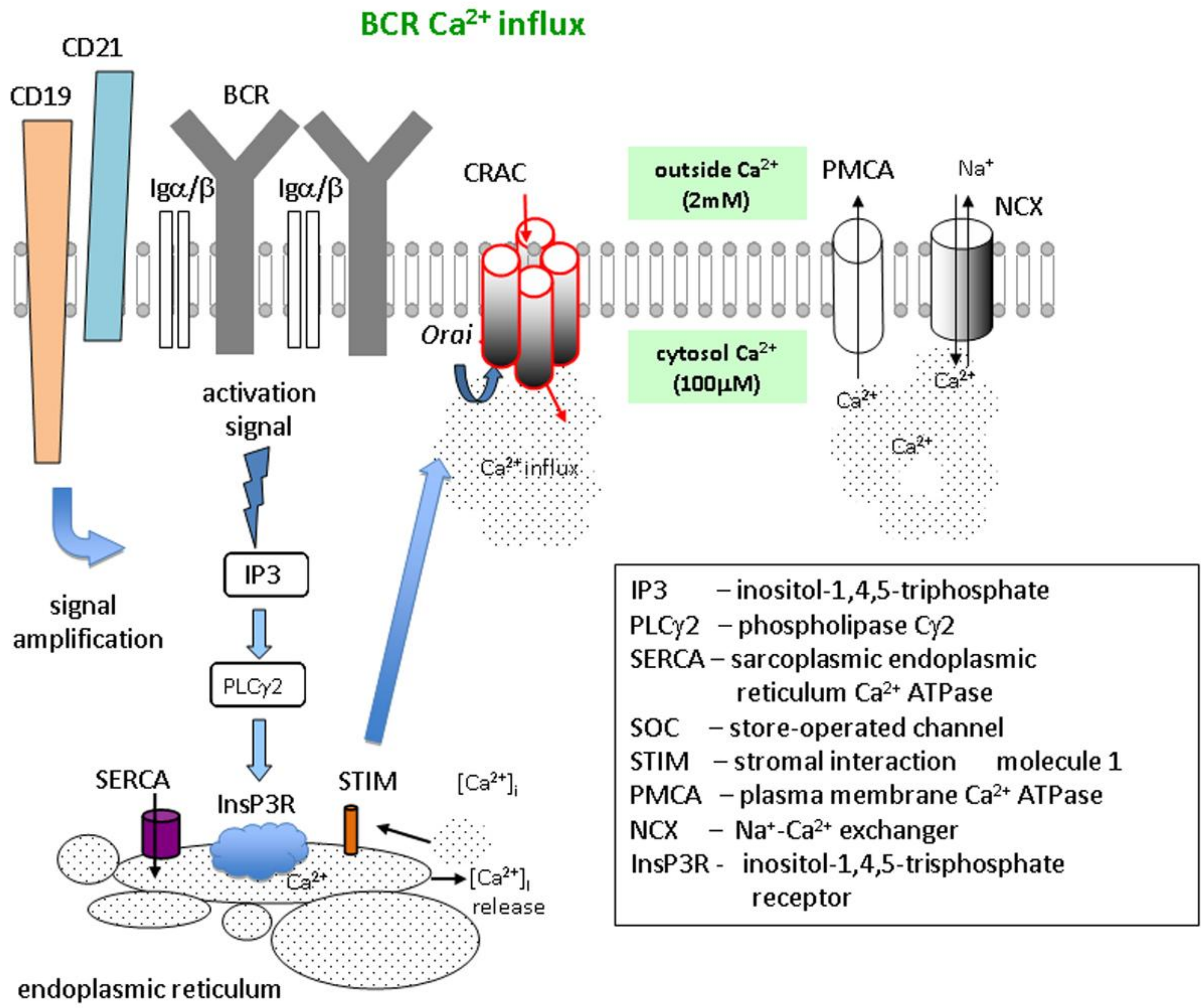

**FIGURE 1.** BCR-mediated $Ca^{2+}$ influx. Upon ligation/cross-linking with an antigen, the BCR signaling complex (IgM/Igα/Igβ/CD21/CD19) elicits an activation signal mediated by IP3, PLCγ2, and InsP3R, leading to rapid depletion of ER $Ca^{2+}$ stores. $Ca^{2+}$ released from the stores activates a specific type of transmembrane $Ca^{2+}$ channel (CRAC) via STIM/*Orai* coupling and initiates $Ca^{2+}$ influx from the extracellular environment. PMCA and NCX represent additional regulatory mechanisms for $Ca^{2+}$ homeostasis.

treatment with nonselective $Ca^{2+}$ ionophores[55]. BCR-dependent $Ca^{2+}$ influx through DAG-activated $Ca^{2+}$ entry channels was recently reported, and this mechanism involved TRPC3 as both a $Ca^{2+}$-permeable channel and a protein scaffold at the plasma membrane for downstream protein kinase Cβ (PKCβ) activation in B cells. In anti-insulin, specific B-cell BCRs that are occupied by autologous insulin were shown to deliver signals that induce changes in intracellular $Ca^{2+}$ mobilization and maintain tolerance by preventing activation of key transcription factors such as NFAT (nuclear factor of activated T cells)[60].

Recent studies have also addressed the role of $Ca^{2+}$ signaling in B-cell gene expression. It was demonstrated that cytoplasmic $Ca^{2+}$ oscillations have pronounced effects on lymphocyte gene expression/transcription factor activation profiles[7,61], and that distinct activating or inhibitory signaling events resulting from BCR engagement trigger qualitatively different downstream nuclear signals[62]. In particular, transient $Ca^{2+}$ fluctuations appeared to regulate the translocation of nuclear factor κB (NF-κB) into the nucleus, while continuous $Ca^{2+}$ increases stimulated the recruitment of NFAT through different molecular mechanisms. The NF-κB–mediated mechanism is well characterized in B cells and involves phospholipase Cγ 2 (PLCγ2) and Bruton's tyrosine kinase (Btk) as intermediaries[63,64,65]. The activation of the NFAT pathway was shown to involve PKCδ and Ras GTPases[66]. Myc transcription factors were also implicated in B-lymphocyte proliferation and differentiation through the amplification of $Ca^{2+}$ signals concurrent with the expression of Myc- and $Ca^{2+}$-regulated target genes[67]. Notably, the exploration of the NFAT signaling pathway in lymphocytes with genome-wide single nucleotide polymorphism (SNP) mapping and RNAi screening led to the identification of *Orai1* as a pore subunit of the CRAC channel[68].

However, despite many informative studies, the nature of $Ca^{2+}$ signaling in mammalian B cells following BCR ligation and the mechanism of influx have not been characterized to the same extent as in T cells.

## SOURCE OF $Ca^{2+}$ INVOLVED IN PROTEIN PHOSPHORYLATION EVENTS AND THE ROLE OF STORE-OPERATED INFLUX

Antigen receptor–induced phosphorylation of protein kinases and phosphatases is required for activation of the BCR-mediated $Ca^{2+}$ signaling pathway. In the absence of kinase phosphorylation, molecular pathways linking BCR ligation/activation to IP3 production are not functional because the phosphorylation and activation of PLCγ, which results in IP3 production, does not occur (reviewed in (Kurosaki[69]). In general, phosphotyrosine (pTyr) activity in lymphocytes is thought to precede the increase in intracellular $Ca^{2+}$ concentration[8,55,70,71,72] resulting from influx through SOC triggered by the release of relatively small quantities of $Ca^{2+}$ from intracellular stores. However, the source of $Ca^{2+}$ involved in the initial signaling protein phosphorylation is not well defined. It has been demonstrated that many initial BCR-triggered $Ca^{2+}$-dependent Tyr phosphorylation events involve primarily $Ca^{2+}$ released from intracellular stores and do not depend on the extracellular influx[13]. This suggests a different role for this phase of $Ca^{2+}$ influx. However, specific signaling molecules that become phosphorylated distinctly before or after the initiation of transmembrane $Ca^{2+}$ influx are yet to be identified with a large-scale phosphoprotein array analysis. Other studies[73] have confirmed the interdependence between $Ca^{2+}$ signaling and protein phosphorylation, in particular that the $Ca^{2+}$ and reactive oxygen intermediates generated upon BCR activation can engage in a cooperative interaction that amplifies early signaling events.

The presence of two distinct types of $Ca^{2+}$-mediated protein phosphorylation events (those that utilize $Ca^{2+}$ released from intracellular stores and events relying on $Ca^{2+}$ brought in by the transmembrane influx) raises a possibility for the following previously unanticipated regulatory role of $Ca^{2+}$ in B-cell activation. Initial $Ca^{2+}$ increases resulting from the depletion of intracellular stores typically are significantly lower (~10-fold) than those caused by the transmembrane influx. It is possible that the low store-derived $Ca^{2+}$ levels primarily accompany only the initial signaling events that are involved in cell activation, while the significantly higher postinflux $Ca^{2+}$ levels that occur later may play an additional role in the inhibitory signaling cascades that down-regulate BCR activation.

## $Ca^{2+}$ SIGNALING IN THE IMMUNE SYNAPSE AND MULTIFACETED $Ca^{2+}$ RESPONSES WITHIN A SINGLE CELL

The immune synapse is an important regulatory structure in B cells that transiently forms on the cell membrane during antigen processing and plays a key role in antigen presentation, interactions between different cells of the immune system, determining signaling thresholds through the engagement of coreceptors, and regulating localized increases of antigen density on the cell surface. Recent advances in the characterization of molecular mechanisms of immune synapse signaling are reviewed in Harwood and Batista[74].

Studies of the relationship between $Ca^{2+}$ signaling and immune synapse dynamics demonstrated that $Ca^{2+}$ signaling peak precedes the formation of central Super Molecular Activation Cluster (cSMAC) by estimated 300 sec[13]. This is consistent with other reports that found the immune synapse playing a down-regulatory role in NK and T-lymphocyte activation after the initial receptor engagement[75,76].

Recent reports have validated an important regulatory role of highly localized signaling events occurring within subcellular structures (microclusters)[77,78,79]. The role of $Ca^{2+}$ in these processes in B cells during antigen response has not been fully investigated. Results published by our group[13] have revealed distinct synapse-related cytoplasmic $Ca^{2+}$ gradients: $Ca^{2+}$ levels in areas adjacent to the immune synapse were different from those in the rest of the cytoplasm, indicating that membrane $Ca^{2+}$ channels in B cells may preferentially relocate (or be more active without relocation) to the area of the cell contacting a corpuscular/directional stimulus. Furthermore, in B cells that were forming multiple synapses (processing several antigen particles simultaneously), localized synaptic $Ca^{2+}$ levels were also different probably due to different stages of signaling in the immune synapse areas. It is our interpretation that at later stages of synaptic signaling, the localized cytoplasmic $Ca^{2+}$ levels were lower because the signaling complex has already assembled and $Ca^{2+}$-dependent signaling initiation events have been completed; in contrast, at the early stages of cell/antigen interaction, $Ca^{2+}$ levels are high in the area adjacent to the contact site (Fig. 2). Other reports also suggested that distinct areas within the SMAC (central part or cSMAC) act as a platform for immunoreceptor internalization and signaling termination[80].

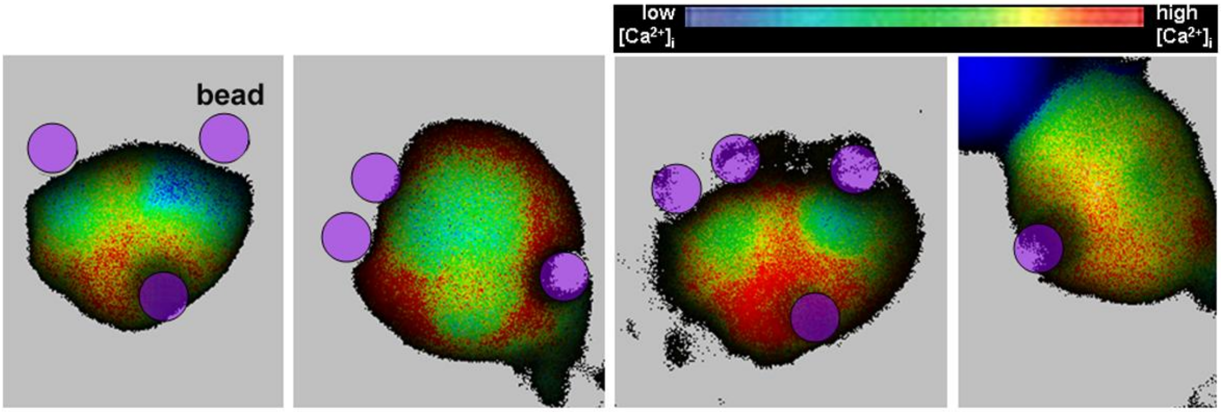

**FIGURE 2**. Heterogeneities in cytosol $Ca^{2+}$ in B cells activated by anti-IgM–coated beads[13]. Examples of cytoplasmic $Ca^{2+}$ gradients in murine K46μ B cells responding to a polarized stimulus. In these experiments, anti-IgM–coated beads (outlined in purple) were brought into contact with K46μ cells labeled with intracellular $Ca^{2+}$ probe Fura-2AM and adhered to the bottom of microscopic dish. Static images (λ340/380 nm ratio; random time points <20 min) demonstrate distinct high- and low-$Ca^{2+}$ areas within the cytoplasm, reflecting different stages of signaling in the immune synapse area (beads were contacting B cells at random, thus interaction times were different). Each panel shows a single cell (40× magnification; cells are approximately 30 μm in diameter).

These observations suggest the presence of a highly localized mechanism that enables compartmentalized regulation of $Ca^{2+}$ levels within the cytoplasm and, as a result, a cell is able to process several corpuscular antigenic stimuli simultaneously and independently. This mechanism may have important implications for “multifaceted” responses, whereby a B cell that is not terminally differentiated is able to balance multiple stimuli of different antigen affinities/avidities and eventually select a “preferred” stimulus to develop a specific response against (based on BCR signal strength and timing, contributions from other signaling pathways through the involvement of coreceptors and other factors). A possible caveat to these conclusions is that intracellular $Ca^{2+}$ gradients may appear as an imaging artifact due to intracellular granules having $Ca^{2+}$ levels different from those in the bulk cytosol, as was described in cytotoxic T lymphocytes rich with acidic lytic granules[81]. However, B cells are not likely to contain nearly as much acidic granules as cytotoxic T cells. Furthermore, imaging artifacts that may appear as intracellular $Ca^{2+}$ gradients due to different $Ca^{2+}$ levels in the nucleus, intracellular granules, or other organelles ought to be distributed randomly (i.e., without any relation to the immune synapse site), which was not the case in the experiments shown in Fig. 2. Noteworthy, studies performed in cytotoxic T-cell lines demonstrated that $Ca^{2+}$ channels do not cluster in the synapse area and are distributed uniformly throughout the cell surface during the first few seconds of signaling[81]. These data, however, were collected at much later time points (minutes into the synapse formation), which may account for the differences in results.

The presence of dynamic high- and low-$Ca^{2+}$ areas within the immune synapse as well as larger-scale differences in $Ca^{2+}$ dynamics in bulk cytosol vs. synapse area in a B cell responding to a localized antigen stimulus are likely indicative of highly localized $Ca^{2+}$-dependent signaling events taking place in subcellular microclusters. One potential experimental approach to further investigate these findings would rely on the development of calmodulin/FRET-based $Ca^{2+}$ sensors[79] targeted at molecules that participate in the formation of the immune synapse in B cells (Ig$\alpha$/$\beta$, CD19, CD21). Such molecule-targeted biosensors can be used for real-time monitoring of $Ca^{2+}$-dependent signaling events in the immediate proximity of molecular signaling microsites. In addition, the heterogeneities in BCR-triggered subcellular $Ca^{2+}$ levels are likely to correlate with localized $Ca^{2+}$-dependent Tyr phosphorylation events.

## ROLE OF CORECEPTORS IN BCR $Ca^{2+}$ SIGNALING

In addition to IgM and adjacent Ig$\alpha$/$\beta$ molecules, the BCR signaling complex engages many additional regulatory coreceptors, such as CD19, CD21, CD22, CD81[82,83]. One major innate immunity factor known to influence B-cell responses is complement, particularly the C3dg protein, which is one of the cleavage products of the C3 component. Invasive pathogenic organisms spontaneously bind and fix complement. C3dg is a ligand for Complement Receptor 2 (CR2, also known as CD21) on the B-cell surface, and CR2-C3dg interaction greatly amplifies signals elicited by the binding of C3dg-attached antigen with its specific B-cell receptor (BCR)[40,82,84]. The complement-mediated signaling pathway can overcome the intracellular inhibitory mechanism responsible for maintaining clonal anergy in B cells. Spontaneous Ab-independent complement deposition on self-tissues and mimetic bacterial pathogens may be an important factor that can disrupt the regulatory mechanism responsible for keeping the potentially harmful autoreactive B cells from responding to self-antigens. One important characteristic of this mechanism is that, unlike BCR ligation with an Ag alone, a BCR/CR2-mediated response involves significantly lower (up to 1000-fold) doses of antigen and does not activate negative signal regulators, such as SHIP-1, SHP-1, SHP-2, and CD22, in order to elicit a comparable response[40]. This contributes to sustained complement-mediated B-cell activation due to lesser impact of signal inhibitors.

A number of studies focused on $Ca^{2+}$ transport and signaling in B cells, and placed a special emphasis on the PLC$\gamma$2-IP3–mediated mechanism of BCR and its coreceptor (CD19, CD21) $Ca^{2+}$ signaling, as well as novel mechanisms for the regulation of cytosolic $Ca^{2+}$ concentration. It has been discussed how these combined inputs could have an impact on the $Ca^{2+}$-dependent regulation of NFAT and NF-$\kappa$B transcription factor pathways, and influence cell-fate choice during humoral immune responses (reviewed in Scharenberg et al.[85]). Different B-cell subsets may differentially modulate $Ca^{2+}$ signaling to control B-cell fate and our previous findings demonstrated substantial differences in $Ca^{2+}$ responses triggered by the engagement BCR[40] as well as BCR coreceptors, such as CD21[86], in B-cell subsets (mature, immature, marginal zone, B-1/B-2 cells) and effects on B-cell development. Recent reports[87] have also indicated that CD21-enhanced $Ca^{2+}$ signaling plays a major role in overcoming B-cell anergy and triggering antigen-specific Ab responses in a subset of anergic B cells.

## $Ca^{2+}$ SIGNALING AND MAINTENANCE OF B-CELL TOLERANCE AND CLINICAL SIGNIFICANCE OF B-CELL $Ca^{2+}$ SIGNALING

Alterations in $Ca^{2+}$ signaling are recognized as one of the factors contributing to the loss of immune tolerance and anergy in B cells. Tolerized/anergic B cells have elevated baseline $Ca^{2+}$ levels and sharply reduced response to BCR ligation[87,88], and this effect is not due to inactivation of STIM1[89]. Current consensus is that the high baseline intracellular $Ca^{2+}$ concentration, which is characteristic of many experimental models of B-cell anergy, is due to the constant signals induced by the self-antigen binding[90], although it is not the case for some transgenic models[88]. Naïve B cells undergoing their initial response to antigen exhibit a rapid rise in intracellular $Ca^{2+}$, but within a few minutes, levels fall to a plateau, which is maintained as long as antigen is present. This plateau level is equivalent to the high baseline of anergic cells. Thus, anergic cells are the physiologic equivalent of chronically antigen-stimulated naïve cells.

Modulating BCR-mediated $Ca^{2+}$ signaling mechanisms is a promising approach to treatment of B-cell–related immune disorders. For example, it was shown that 1,4-benzodiazepine Bz-423 extends the rise in intracellular $Ca^{2+}$ that accompanies anti-IgM stimulation, and this effect mediates the synergistic death response. Because hyperactivation and altered $Ca^{2+}$ signaling are distinguishing features of autoreactive lymphocytes in autoimmune diseases such as lupus, Bz-423 is believed to preferentially target disease-causing cells for apoptosis on the basis of their activation state[91]. Also, $Ca^{2+}$-activated neutral proteases (calpains) that become active in cells responding to signals inducing a rise of cytoplasmic $Ca^{2+}$ are involved in the regulation of apoptosis of some cell types by interaction with caspase-3 and have been shown to play a role in B-cell survival[92].

Studies in human B cells that examined the role of extracellular $Ca^{2+}$ sensing in promoting cell activation[93] have determined that responses to changes in extracellular $Ca^{2+}$ levels activated PI3 kinase/AKT, calcineurin, ERK, p38 kinase, PKC, $Ca^{2+}$/calmodulin kinase II, and NF-κB signaling pathways, and resulted in transcription of the early response gene, *CD83*. This extracellular $Ca^{2+}$-sensing mechanism was also shown to enhance B-cell responses to TLR, BCR, and cytokine receptor agonists. These results may indicate a mechanism by which B cells prepare to engage in immune responses by responding to $Ca^{2+}$ fluctuations in their environment.

Clinical aspects of B-cell $Ca^{2+}$ signaling are being investigated with increasing appreciation. For example, recently reported effects of Rituximab (anti-CD20 mAb) on BCR signaling revealed inhibition of the signaling cascade involving Lyn, Syk, PLCγ2, Akt, and ERK, and $Ca^{2+}$ mobilization. This inhibitory effect correlated with a decrease of raft-associated cholesterol, complete inhibition of BCR relocalization into lipid raft microdomains, and down-regulation of BCR immunoglobulin expression[94]. Impairment of $Ca^{2+}$ signaling was shown to silence CD19 expression in primary pre-B cells by down-regulating CD19 gene expression upon pre-BCR activation through inhibition of the E2A transcription factor by $Ca^{2+}$/calmodulin[95]. Another calmodulin-related protein, Pcp4, a molecule that modulates $Ca^{2+}$ by binding to calmodulin via an IQ motif, was decreased in anergic and CD21-negative B cells[96,97,98]. BCR-mediated $Ca^{2+}$ signaling was also impaired in B cells from patients with common variable immunodeficiency. Although in this instance proximal BCR signaling events were normal, including normal PLCγ2 phosphorylation and $Ca^{2+}$ release from intracellular stores, $Ca^{2+}$ influx was significantly impaired. CD22, a negative regulator of $Ca^{2+}$ signals in B cells, is highly expressed on $CD21_{low}$ B cells in these patients and therefore might be involved in the attenuated $Ca^{2+}$ response of this B-cell subpopulation[99].

Overall, this mini-review tried to highlight several relatively underexplored aspects of $Ca^{2+}$ signaling unique to B-lymphocyte responses to both antigen-specific and nonspecific stimuli. These areas are being actively studied to obtain further insights into the nature and molecular details of $Ca^{2+}$ signaling in B lymphocytes.
